# Supplementary material for: Loss of Continuity of Care in Pediatric Neurology Services during COVID-19 Lockdown: An Additional Stressor for Parents
Source: Children (Basel). 2022 Jun 10;9(6):867. doi: 10.3390/children9060867 (PMC9222060; doi:10.3390/children9060867)
Supplement: Supplementary file 1 [file children-09-00867-s001.zip › children-1707956-supplementary-done.pdf]

children

MDPI

The floor to parents. The impact of Covid-19 pandemic on the care of children with neurological diseases.

Dear parent, the COVID-19 health emergency has had and continues to have an impact on many aspects of our daily lives. Isolation and social distancing, necessary to stem the spread of the new Coronavirus, have limited access to various services essential for the physical and mental well-being of children and their families such as school, meeting places and hospitals. This is even more true for those families who need the support of services for the care and treatment of their children suffering from neurological diseases. We would like to give parents a voice with a study, through an anonymous questionnaire, to understand their greatest difficulties in this time and to be able to identify protective factors and risk factors. The research will help clinicians to understand the impact of the Covid-19 pandemic on the care of children with neurological diseases. In addition, it will help us to adapt the health response to families, in an overview, focused not only on the treatment of the disease but on the care of the person.

*Required field

The floor to the parent. The impact of the Covid-19 pandemic on the care of children with neurological diseases.

YOUR PRIVACY IS IMPORTANT TO US. *

The undersigned acquired information regarding the purposes of the collection of data provided with this questionnaire with the information above and aware that the processing will concern "particular personal data referred to in art. 9 of the GDPR" CONSENTS to the processing of data necessary for the performance of the research.

Yes

No (by ticking this option you will not be able to participate in the search)

GENERAL DATA

Age of your child *

SEX *

Male

Female

Disease affecting your child *

Psychomotor delay

Autism

Epilepsy

Infantile cerebral palsy

Genetic-Metabolic disease

Other:

If the answer is "Other", specify

Who fills out the questionnaire? *

Mom
Dad
Other:

If the answer is "Other", please specify.

Mom education level

Elementary school
Lower secondary school
2/3 years of high school
High school
A few years of university
Courses/ Post-university specialization

Dad education level

Elementary school
Lower secondary school
2/3 years of high school
High school
A few years of university
Courses/ Post-university specialization

Mom job

Dad Job

Has the lockdown affected your workload? *

Yes
No

If so, how?

homeworking
Reduction of working hours
Layoffs
Dismissal
Temporary suspension of work activity (freelancer, catering, bar, etc.)
Other:

If the answer is "Other", please specify.

Your home *

has a balcony
has a condominium terrace
has a terrace
there is a garden
none of the above

COVID-19 and Lockdown

Has your child been affected by Covid-19 (assessed by a suitable diagnostic method)? *

Yes

No

If so, which symptoms occurred?

Cough

Fever

Respiratory difficulties

Rash

Conjunctivitis

Gastrointestinal symptoms

No symptoms (positive swab performed for contact at risk)

Other:

If the answer is "Other", please specify.

Have there been cases of COVID 19 among your relatives? *

Yes

No

Have there been cases of COVID 19 among your friends/acquaintances? *

Yes

No

Has your child been able to attend school remotely via online platforms? *

Yes

No

If so, how do you judge the experience?

Totally negative

0

1

2

3

4

Totally positive

What benefits did you notice? *

He was able to continue the educational activities

Was able to maintain contact with teachers or schoolmates

He was able to keep busy at a time when he was forced to stay at home.

Other:

If the answer is "Other", please specify.

What problems or negative experiences have there been? *

Not availability of sufficiently efficient internet connection

Unfamiliarity on the part of your child with the use of the necessary technologies

Inability to use the necessary technologies due to your child's clinical and neurological condition

Inadequacy of the technological means for the maintenance of the usual interpersonal relationships with teachers and / or companions
Other:

If the answer is "Other", please specify.

During the lockdown period, have health/welfare/rehabilitation services normally provided to your child been suspended? *

Yes
No

If so, the lack of which performance has weighed, in your opinion, more on the clinical and general picture of your child?

Periodic clinical/instrumental/laboratory checks at the reference centre
Contact with the treating specialist
Checks at the pediatrician or attending physician
Home care (nursing and non-nursing)
Rehabilitation/neuropsychomotricity activities
Other:

If the answer is "Other", please specify.

During the lockdown period, have you been able to easily contact the specialist or the care team of your child? *

Yes
No

If so, how?

By phone
By mail
Video call
Face-to-face visit

Did your child need urgent health services during the lockdown period? *

Yes
No

If so, why?

Reason related to his neurological pathology
Reason unrelated to his neurological pathology

How was it dealt with? *

Pediatrician home visit/mmg
Pediatrician outpatient visit /mmg
Urgent outpatient visit referral specialist
Access in PS
Hospitalization

During the lockdown period, did your child have access to rehabilitation/neuropsychomotor services remotely? *

Yes

No

If so, how do you judge the experience?

Totally negative

0

1

2

3

4

Totally positive

What benefits did you notice?

He was able to continue without interruption the activities previously carried out with positive effects on his clinical situation

He was able to maintain contact with his therapist or with the team responsible for his rehabilitation project

It allowed a greater parental involvement in the rehabilitation activities because there was a need to mediate the instructions provided remotely by the staff

Other:

If the answer is "Other", please specify.

What problems were there?

Insufficient internet bandwith

Ineffectiveness of activity carried out because it is based on instructions provided by the therapists and not on the direct activity

Ineffectiveness of the computerized systems to mediate interpersonal relationships with the therapist

Inability to use the technological instrumentation by my child due to his neurological condition

Unfamiliarity of child or parent with the use of the necessary technologies

Other:

If the answer is "Other", please specify.

About the underlying pathology, during the lockdown period the clinical condition of your child: *

had improved

remained stable

has gotten worse

From cognitive and ability point of view, during the lockdown period, your child: *

has improved

has gotten worse

has remained stable

From a behavioral point of view, during the lockdown period, your child: *
has improved
has gotten worse
has remained stable

Lockdown: the parents' perspective.

We now ask you to complete the Perceived Stress Scale (PSS; Scale for Perceived Stress). This scale is the most widely used tool for measuring the perception of stress. It is a measure of the degree to which situations in a person's life are evaluated as stressful. PsS questions are about feelings and thoughts about the last month. Imagine these questions referring to the entire Lock-down period. For each item, you are asked to indicate how often they felt in a certain way, in the following way:0 = Never 1 = Almost never 2 = Sometimes 3 = Quite often 4 = Very often

In the last month, how often have you felt out of yourself because something unexpected has happened? *
0
1
2
3
4

Over the past month, how often have you had the feeling that you are unable to have control over the important things in your life? *
0
1
2
3
4

In the last month, how often have you felt nervous or "stressed"? *
0
1
2
3
4

Over the past month, how often have you felt confident about your ability to handle your personal problems? *
0
1
2
3
4

In the last month, how often have you had the feeling that things were going as you said? *
0
1
2
3
4
In the last month, how often did you feel like you couldn't keep up with all the things you had to do? *
0
1
2
3
4
In the last month, how often have you felt that you are able to control what irritates you in your life? *
0
1
2
3
4
In the last month, how often have you felt you have mastered the situation? *
0
1
2
3
4
Over the past month, how often have you been angry about things that were out of your control? *
0
1
2
3
4
In the last month, how often did you have the feeling that difficulties were accumulating to such an extent that you could not overcome them? *
0
1
2
3
4

Thank you so much for cooperating with us.
